# Mechanism of Stone (Hardened Endocarp) Formation in Fruits: An Attempt toward Pitless Fruits, and Its Advantages and Disadvantages

**DOI:** 10.3390/genes13112123

**Published:** 2022-11-15

**Authors:** Muhammad Khalil Ullah Khan, Noor Muhammad, Zhuolong Jia, Jianying Peng, Mengjun Liu

**Affiliations:** 1 College of Horticulture, Hebei Agricultural University, Baoding 071001, China; 2 Center of Chinese Jujube, Hebei Agricultural University, Baoding 071001, China

**Keywords:** stone fruit, lignin, endocarp, molecular regulation

## Abstract

Stone (hardened endocarp) has a very important role in the continuity of plant life. Nature has gifted plants with various seed protection and dispersal strategies. Stone-fruit-bearing species have evolved a unique adaptation in which the seed is encased in an extremely hard wood-like shell called the stone. The lignification of the fruit endocarp layer produces the stone, a feature that separates drupes from other plants. Stone cells emerge from parenchyma cells after programmed cell death and the deposition of cellulose and lignin in the secondary cell wall. Generally, the deposition of lignin in primary cell walls is followed by secondary thickening of cell walls to form stone cells. This review article describes the molecular mechanisms and factors that influence the production of stone in the fruit. This is the first review article that describes the molecular mechanisms regulating stone (harden endocarp) formation in fruits. This article will help breeders understand the molecular and genetic basis for the stone formation in fruit, and this could lead to new and innovative directions to breed stoneless fruit cultivars in the future.

## 1. Introduction

**Figure 1:**
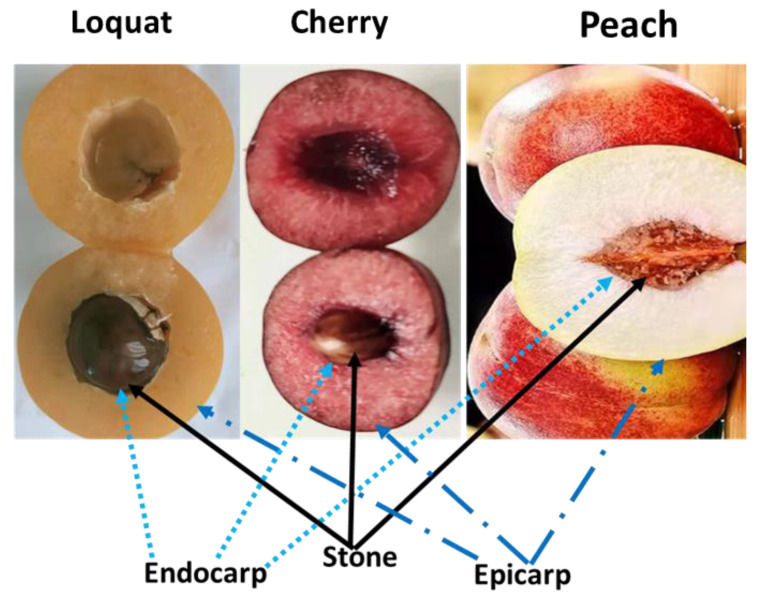
The fruit of loquat, cherry, and peach show their different parts: epicarp, endocarp, and stone.

Stone fruits, also known as drupes, are a group of plants, the majority of which belong to the genus *Prunus* L., which is a member of the Rosaceae family [1,2], that provide a nutrient-rich source for the human body [3]. Stone fruits get their name from the woody endocarp (stone or pit) that characterizes these species’ fruits. The fleshy epicarp and mesocarp, which envelop the stony endocarp, are the edible parts of stone fruits, as shown in Figure 1. Almonds and certain apricots, whose seeds are consumed, are exceptional cases. These fruits include apricots, cherries, and numerous species of commercially important plums, peaches, and nectarines [2,4].

This review article discusses the molecular mechanisms and factors that contribute to stone formation in fruit. This is the first review article that explains the molecular mechanisms that control stone formation (hardened endocarp) in fruits. This article will aid breeders in recognizing the molecular and genetic basis for stone formation in fruit, potentially leading to new and innovative breeding strategies for stoneless fruit cultivars in the future.

## 2. Development and Structural Features of the Stone Fruit

All stone fruits include a fruit wall (pericarp) that typically contains a single seed [2]. The fruit wall is formed from the ovary and is made up of three layers: the stone is enclosed by the mesocarp, which is made up of flesh (endocarp) (Figure 1).

Generally, in all fruits, the pericarp is a group of tissue layers arising from the carpel ovary [5]. Thus, the pericarp in fleshy fruits is frequently divided into three layers: the endocarp (innermost layer), the mesocarp (middle layer), and the exocarp (skin or outer layer) [5]. The mesocarp is the soft edible part among most fleshy fruits (Figure 1). In some cases, the fleshy fraction is developed from tissues besides the ovary; these are sometimes referred to as false fruits. Apple, for example, yields a pome fruit wherein the center reflects the real ovary-derived fruit, and the edible portion arises from the hypanthium, which is produced by the stitched base of petals and sepals [5]. The endocarp is the tissue layer located close to the seed that is distinguished from the inner layer of the ovary. It has a variety of functions in fruit and can be fleshy like watermelon, fibrous like mango, or extremely hard and durable like a peach. Drupes are fruits with a hardened endocarp. Ber, peach, cherry, plum, almond, coffee, mango, olive, coconut, pistachio, date, raspberry, oil palm, and walnuts are examples of drupes [5,6]. Studies indicate that the evolution of the endocarp’s woody structure is caused by straightforward genetic changes in a small number of genes that regulate growth [2,5].

## 3. Molecular Basis for the Configuration of Endocarp

The characterization of the underpinning genes and signaling pathways that regulate the differentiation of ovarian tissues into endocarp, mesocarp, and exocarp is being accelerated by breakthroughs in genetics and technological innovations in genomics. Studies in Arabidopsis are leading the way, and the acquired knowledge is now being applied to a variety of other plants [6,7]. Although our current understanding is still limited, it is becoming clear that the same or closely related cellular programs make a significant contribution to pericarp tissue differentiation across species.

The *Prunus* appears to contain only drupes, such as peaches, plums, apricots, almonds, and cherries, which produce a large, lignified endocarp that envelops the seed and is usually referred to as the stone. These fruits develop in a sigmoidal structure, with a pause in growth that corresponds with endocarp strengthening and hardening [7,8]. This could be due to the enhanced carbon and energy requirements related to lignification [9]. Evidence from some studies into the pattern and timing of endocarp lignification has revealed that it is a great collaborative process that takes place over 2 to 3 weeks [7,10].

Whereas the duration varies by cultivar, lignin is usually perceptible 30–45 days after bloom in a slender endocarp layer all along the fruit suture and the funiculus. However, after a few days, the complete endocarp starts to lignify. Because the tissue in which lignin is first distinguishable is also the first to harden, hardening appears to follow a similar pattern as lignin accumulation [5]. Since molecular studies are still lacking, expression profiling implies that many of the same genes that regulate endocarp progression in other species also control endocarp development in *Prunus persica* [7]. The *SHP* (*SHATTERPROOF*) and *STK* (*SEEDSTICK*) homologs in peaches were discovered to be upregulated in the endocarp after pollination. *SHP* and *STK* expression was limited to the endocarp and seed but began to decline near the initiation of lignin deposition. Similarly, *FUL* (*FRUITFUL)* expression continued to remain higher in the mesocarp and exocarp but was low in the endocarp [5,8]. This is consistent with a potential role in endocarp lignification margin demarcation. Following the decline of *SHP* and *STK* expression, the expression of a peach *NST1* (NAC secondary wall thickening promoting factor1) homolog, as well as secondary metabolism and cell wall biosynthesis genes, increased rapidly [6]. Although there were no clear homologs of *ALC (ALCATRAZ)* and *IND* (*INDEHISCENT*) in peach, the two most similar genes were not endocarp specific [7]. Tani et al. [11] demonstrated that the expression profiles of peach *SPT* (streptomycin phosphotransferase) were consistent with a role in endocarp margin specification [7]. These suggest that highly similar pathways likely control pericarp development in *Prunus* [5].

Flavonoids, such as lignin, are produced through secondary metabolic pathways that are considered to be able to compete with lignin because they both use the same phenylpropanoid pathway precursors [8]. During early fruit development, the lignin and flavonoid pathways are both activated in peach fruit [5,7,12]. Such actions are spatially organized, resulting in the induction of phenylpropanoid pathway genes in all three pericarp layers: endocarp, mesocarp, and exocarp (though to a much greater degree in endocarp). However, in the endocarp, this upregulation is preceded by lignin pathway induction and flavonoid pathway repression, whereas in the mesocarp and exocarp, flavonoid pathway genes are induced while lignin pathway genes are suppressed [5].

This coordination, it is assumed, allows the fruit to accrue defense compounds, flavor, and color progression in the mesocarp and exocarp, while also allowing endocarp lignification. Thus, endocarp lignification appears to be linked to the synthesis of compounds required for defense, herbivore appeal, and seed dispersal [13]. *Prunus* endocarp traits that have been chosen through the breeding show a great deal of variation. Almond shells, for example, differ in terms of endocarp thickness, hardness, and fragility. These agronomic characteristics are essential for the processing of almonds and other types of nuts. Some peach varieties have a phenotype known as “split pit”, which occurs when the endocarp fails to enclose along the suture, leaving the seed vulnerable to disease [5]. Split pits are more common in peach cultivars which proceed with fast fruit growth before the stone has fully solidified. Tani et al. [10] discovered that *SHP* expression is relatively low in a split pit-resistant variety during the lignification stage, whereas *FUL* expression is significantly higher in the sensitive variety later in fruit development [5].

Moreover, “Stoneless” is a naturally occurring phenotype discovered in Sans Noyau, a wild-type plum species from France [9]. The endocarp layer of “Stoneless” does not develop properly, resulting in a partially exposed, uncovered seed that sits within an empty fruit aperture. Callahan et al. discovered that the “Stoneless” phenotype is highly affected by the environment, as fruits in hot spring temperatures seem to have a more complete stone, whereas fruits in cooler spring temperatures have very little stone [9]. The remaining hardened tissue in “Stoneless” appears to correspond with the funiculus and a portion of the placental endocarp wall [9]. Because secondary metabolic genes are still induced, expression studies indicate that the lignification process is likely to function normally in “Stoneless.” The absence of endocarp tissue demonstrates that this mutant lacks a full endocarp layer [5].

## 4. Molecular Regulation of Stone Formation in Fruits

The endocarp and lignin are the two main factors responsible for the stone formation in fruit. The molecular mechanisms responsible for the endocarp and lignin deposition are described as follows.

### 4.1. Lignin

The majority of the enzymes and regulating steps of the lignin biosynthesis route (phenylpropanoid pathway), an aromatic polymer that is commonly found in the secondary walls of plants, have been characterized [5,6]. Lignification is a complex process that occurs primarily in higher plants and has the primary goal of strengthening the structural stability of the plant’s vascular system and building a barrier against diseases and pests [14]. The production, transfer, and deposition of lignin are all important in the growth of stone [15,16]. Lignin is a three-dimensional non-crystalline polymer with complex network architecture. In various plants, and in different areas of the same plant, the dispersion, quantity, and structure of lignin in the cell wall varies greatly [15,17]. In lignin, there are methoxy, hydroxyl, carbonyl, and other groups. The presence and distribution of these functional groups are determined by the type of lignin found in a plant [18]. Lilac (Syringyl) propane, guaiacyl propane, and hydroxyphenyl propane are the three types of structural components found in lignin [18]. Syringyl lignin (S-lignin) is made up of syringyl complexes, guaiacyl lignin (G-lignin) is made up of guaiacyl units, and hydroxyphenyl lignin (H-lignin) is made up of p-hydroxyphenylpropane unit cells. A multitude of random links joins these three structural elements [15].

Several lignin architectures can be created by changing the chemical functional groups and chemical bond properties of lignin [19,20]. Lignin’s main building block is phenyl propane, which is linked together by ether and carbon-carbon bonds. Changes in important bond pairings and variances in chemical functional groups affect lignin’s mechanical qualities and chemical reactivity [18]. Chemical functional groups and chemical bond properties can be studied utilizing spectroscopic techniques to learn more about the lignin biosynthetic route and metabolic regulation of lignin formation [15,19].

The transcriptional regulatory network and endocarp lignification in Arabidopsis have both been thoroughly explored in connection to dehiscence [21]. In the early 1960s, Ryugo [22,23] observed the management of lignin synthesis and build-up in peach stones. In the peach endocarp, lignification is a highly controlled process, as evidenced by following developmental investigations [12]. Furthermore, a transcriptional network controlled by the genes *NAC* (NO APICAL MERISTEM) and *MYB* (myeloblastosis) was discovered in a well-preserved regulatory pathway, which induces the creation of peach endocarps or Arabidopsis dehiscence [6,7], and is crucial for the formation of secondary walls and lignification.

### 4.2. Endocarp

All fruits, whether dry or fleshy, have tissue layers generated from the carpel ovary, which are collectively known as the pericarp [5]. The pericarp is frequently divided into three layers: the endocarp (innermost layer), the mesocarp (middle layer), and the exocarp (outermost layer) (skin or surface layer), as shown in Figure 1. Dry fruit pericarp differentiation can be difficult to spot because each layer has only a few rows of cells [5]. The endocarp is a tissue layer directly close to the seed that differs from the inner layer of the ovary [5]. It can be soft, as in watermelon; fibrous, as in mango; or highly rigid and resilient, as in a peach; and play a variety of roles in fruit functions. Drupes are fruits with a hardened endocarp [5].

*SHP1*, *SHP2*, *STK*, and *FUL* were among the MADs-box genes (MCM1, AGAMOUS, DEFICIENS, and SRF (serum response factor) discovered to play a role in fruit endocarp development [24]. These TFs work in concert with the *IND*, *ALC*, and *RPL* (Ribosomal Protein Large subunit) to promote endocarp development [25,26]. Genes of the phenylpropanoid pathway may be regulated by transcription factors, such as NST1 (NAC secondary wall thickening promoting factor 1), which could have an impact on the endocarp’s growth and lignification in apricot [27].

## 5. Molecular Mechanisms of Endocarp Lignification

The lignified endocarp is a unique characteristic of ripe drupe fruits, but it develops early in fruit development and is lignified in phase II of the double helix fruit-growth curve when mesocarp growth is halted [28]. This cycle of competition for nutrient uptake among fruit tissues and seeds highlights the presence of cyclic activities [29,30,31]. The endosperm expands fast during nucellus absorption during stage II when the endocarp is lignifying, and metabolites stored in the endosperm subsequently stimulate embryo growth [4,31,32]. 

**Figure 2:**
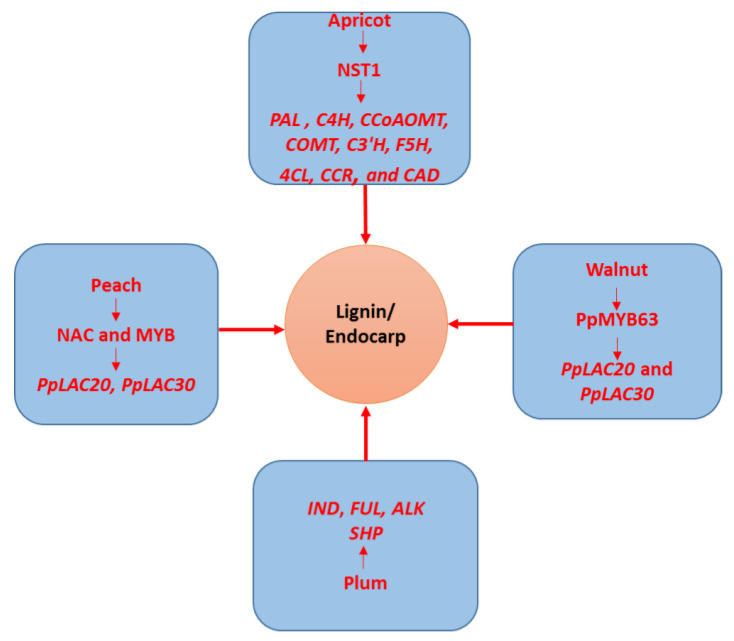
Role of different genes in lignin biosynthesis/endocarp development in fruits. Apricot, peach, plum, and walnut are taken as an example. The NST1 regulates the expression of *PAL, C4H, CCoAOMT, COMT, C3’H, F5H, 4CL, CCR,* and *CAD* genes resulting in endocarp formation. The TFs NAC and MYB activate the expression of *PpLAC20, and PpLAC30* in peach resulting in endocarp lignification. Similarly, in plum fruit, *IND, FUL, ALK, and SHP* regulate the production of stone/endocarp, while PpMYB63 activates the expression of *PpLAC20* and *PpLAC30* genes regulating endocarp development.

The endocarp is also important for maintaining and interacting with developing seeds [5]. Consumers, on the other hand, tend to choose soft-kernel or seedless fruits due to their superior quality and ease of use [33]. Apricots, and other common drupe-producing plants (such as peach, plum, cherry, almond, date, and walnut), produce fleshy fruits with only one carpel [33]. The growth stages of apricot fruit have been identified and classified as follows: S1, the first exponential growth stage; S2, the slow-growing stage; S3, the second exponentially growing state; and S4, the fruit ripening stage [6,33]. The growth pattern of peach endocarp tissue is quite similar to that of apricot [5,33]. Endocarp hardening seems to follow a similar pattern as lignin build-up, as the first tissue to harden is also the first to recognize lignin [5]. As a result, the phenylpropanoid pathway is anticipated to play a crucial role in endocarp lignification. The phenylpropanoid pathway involves several key enzymes, including PAL (phenylalanine ammonia-lyase), C4H (cinnamate-4-hydroxylase), CCoAOMT (caffeoyl-CoA O-methyltransferase), COMT (flavone 3′-O-methyltransferase), C3′H (*p*-coumaroylshikimate/quinate 3′-hydrolxylase), F5H (ferulic acid 5-hydroxylase 1), 4CL (4-coumarate–CoA ligase), CCR (cinnamoyl-CoA reductase), and CAD (Cinnamyl alcohol dehydrogenase) [33]. In a well-conserved regulatory pathway that has been linked to both dehiscence (*Arabidopsis*) and endocarp development, a transcriptional system characterized by *NAC* and *MYB* genes was discovered in peach [7]. By activating this route, this network plays an important role in secondary wall development and lignification [21]. Fruit kernels come in a wide range of shapes. The endocarp of the “split pit” peach, for example, does not enclose along the suture, leaving the seed vulnerable to pests and disease [34], whereas the wild-type *Prunus domestica* ”stoneless” plum produces imprecisely established endocarps that only incompletely encase the seed [9]. Likewise, the apricot tree “Liehe” produces a thin, soft, and cleavable endocarp, making it a one-of-a-kind fruit in China [6]. “Liehe” endocarp composition was much lower than ‘Jinxihong’ endocarp lignin content. Furthermore, the co-expression network and expression pattern identified the 34 genes involved in the phenylpropanoid pathway [33]. *NST1* may impact the lignin deposition of the “Liehe” apricot endocarp by regulating the expression of *CAD* [33]. The role of different genes in lignin biosynthesis/endocarp development in fruits is shown in Figure 2.

Based on phylogenetic, sequence, and expression profiling analyses, Qui et al. [4] stated that peach laccase genes (*PpLAC7*, *PpLAC19*, *PpLAC20*, *PpLAC21*, *PpLAC27*, *PpLAC*28, and *PpLAC30*) may be related to lignin biosynthesis and endocarp hardness in peach fruit. Similarly, *PpLAC20* and *PpLAC30* are most likely important members involved in peach lignin biosynthesis [4]. A peach MYB TF, PpMYB63, a homolog of AtMYB58 and AtMYB63, can activate the *PpLAC20* and *PpLAC30* promoters. Thus, *PpLAC20* and *PpLAC30* are candidates involved in peach lignin biosynthesis, hardening the peach endocarp [4].

Furthermore, laccase genes have a vital role in the lignification of the walnut endocarp, and the walnut laccase gene *JrLAC12–1* has a potential role in the lignification of the walnut endocarp [8]. It is conjectured that IAA (Indole-3-Acetic Acid) has a significant regulatory role in the process of walnut endocarp hardening. The *AUX/IAA* (Auxin/Indole-3-Acetic Acid) genes (*JrIAA9*, *JrIAA16,* and *JrIAA27*) were consistent with that IAA content and could possibly play an important role in walnut endocarp hardening [35].

The differentially expressed genes involved in the phenylpropanoid pathway and flavonoid pathway include *COMT, C3′H, HCT, CAD, POD, C4H, CCoAOMT, CCR,* and *CHS*. These have a potential role in the endocarp development and lignification of walnut [36]. Similarly, the *CCoAOMT* gene plays a key role in regulating the rapid endocarp lignification process in *Davidia involucrata* Baill. [37]. The expression levels of *COMT, C3′H, HCT, CAD, POD,* and *C4H* genes were higher in walnut [36]. TFs (transcription factors) such as *MYB, NAC,* and *LBD* (Lateral organ boundaries domain) also had significantly high expression levels in endocarp development and lignification of walnut [36].

**Figure 3:**
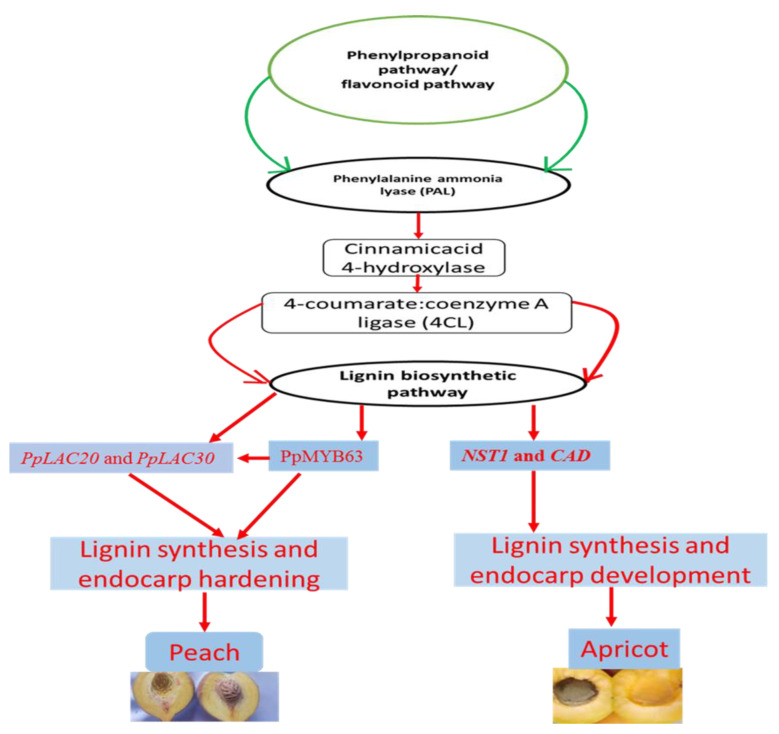
Role of phenylpropanoid/flavonoid biosynthetic genes involved in lignin synthesis and endocarp hardening. The peach and apricot are shown as an example in this figure. The *PpLAC20* and *PpLAC30* are activated by the PpMYB63 transcription factor and are candidate genes involved in peach lignin biosynthesis and hardening of the peach endocarp. The NST1 activates expression levels of CAD (Cinnamyl alcohol dehydrogenase) genes resulting in the synthesis of lignin and endocarp development in the apricot. The role of genes was described recently in peaches by Qui et al. [4] and in apricot by Zhang et al. [33].

Ryugo discovered the presence of lignin in peach stones in the early 1960s [22,23]. Lignin is a plant-only chemical that plays a critical role in tree crops for pulp and paper manufacturing, fodder crops for digestibility, and, more recently, biofuels [7]. Lignin deposition inside specific fruit tissue layers is a common motif in seed preservation and distribution, and it is especially noticeable in *Prunus* stones [7]. Lignification of fruiting structures evolved in certain cases to preserve seeds from disease and stress [7,38]. The *SHATTERPROOF*, *SEEDSTCK*, and *NAC-SECONDARY WALL THICKENING PROMOTING FACTOR 1* was discovered to be exclusively expressed in the endocarp, whereas the negative regulator *FRUITFUL* predominated in the exocarp and mesocarp in peach genes comparable to known endocarp-determinant genes in *Arabidopsis* [7]. The role of phenylpropanoid/flavonoid biosynthetic genes involved in lignin synthesis and endocarp hardening is shown in Figure 3.

Similarly, TFs, such as the *MYB* genes, code for a broad group of transcription factors involved in a variety of biological activities in plants, including lignin production [39,40]. *MYB85, MYB58, MYB63, MYB46, MYB83, MYB20, MYB42*, and *MYB43* have been shown to trigger the expression of monolignol genes in *Arabidopsis* [41,42,43]. *AtMYB15* governs defense-induced lignification and basal immunity by binding directly to the consensus sequences of an MYB-responsive region found in secondary cell wall genes [44]. The *PtoMYB92/PtrMYB3/PtrMYB20* are transcriptional promoters in poplar that promote lignin accumulation during secondary cell wall development [45,46]. Bomal et al. [47] identified *PtMYB1* and *PtMYB8* from *Pinus taeda* as inducers that regulate secondary cell wall deposition in conifers. The *EgMYB2* of *Eucalyptus grandis* co-localizes with a quantitative trait locus for lignin concentration in transgenic tobacco plants, and its overexpression promotes lignin production [48]. The *MYBs* can govern lignin biosynthesis in both vegetative and fruit parts, in addition to regulating lignin biosynthesis in vegetative tissues. The *EjMYB1* and *EjMYB2* of *Eriobotrya japonica*, for example, are activators and repressors, respectively, that regulate chilling-injury-induced fruit flesh lignification through potential interaction with AC elements [ACC(T/A)ACC] in the promoter region of lignin biosynthesis genes [49,50]. Various genes involved in the production of endocarp and lignin are shown in Table 1.

## 6. History of Stoneless Fruit and an Attempt of Making Stoneless or Pitless Fruits

The popularity of fruits, such as grapes, sweet oranges, and watermelon, has risen dramatically as a result of the emergence of seedless varieties of these fruits [9]. Given the marketing value for cultivars without pits and the lower production costs for processed and dried fruit, the economic possibility of doing the same for stone fruit seems encouraging [6,9]. The seed and the stone, a tough, woody coating enclosing the seed, would need to be removed in order to produce stone fruit without pits [9]. In the early 1900s, the pioneering breeder Luther Burbank used a wild-type plum that exhibited some stonelessness to begin working toward the aim of producing stoneless plums [51]. Through breeding, he was able to combine the stoneless characteristic with better fruit quality and even discovered lines lacking seeds, but he was never successful in totally getting rid of the stone or the seed [9,51]. Although Burbank’s quest to revolutionize plum production in this way may have failed, he nevertheless made a significant contribution by proving that the stone can be almost eradicated without compromising fruit quality or quantity. Burbank bred this wild plum with cultivated varieties in California but, because of the inadequate understanding of genetics at the time, he did not disclose segregation ratios, therefore it is unclear if this feature is single- or multi-genic [9,51].

## 7. Advantages and Disadvantages of Stone in Fruits

The endocarp is the deepest layer of the pericarp, which grows from the ovary. Several significant economic fruits, including peach, apricot, plum, almond, cherry, mango, olive, and coffee, depending on the hardened endocarp for seed protection and dispersion [6,7,52,53,54,55]. The extensively lignified endocarp serves as a protective habitat for seed development in plants [6]. A crucial characteristic of mature drupe fruits is the hardening of the endocarp. The development of secondary walls and the accumulation of lignin are the main factors [6]. According to a biochemical study, olive and peach endocarps had significantly more lignin than pine stems [6], indicating that the tissues of fruit endocarps undergo relatively intense secondary wall development. Lignification, also known as lignin accumulation, in the peach endocarp plays significant physiological and evolutionary responsibility in facilitating waterproof shelter against seed water loss during growth and development, avoiding animal digestion of the seeds, and improving seed dispersal.

On the other hand, since fruit trees are vegetatively multiplied and the seeds are not consumed, nuts are normally regarded by the processing sector as waste material and must be removed, usually by burning. Stone clearance and discharge thus significantly increase manufacturing costs and cause pollution [9]. Additionally, the existence of stones and pit pieces in dried and processed fruit is a concern for processing units and may result in item denial, consumer injury, and ensuing legal acts [7,9,27].

## 8. Conclusions and Prospects

In this review article, we highlighted the mechanisms of stone (hardened endocarp) formation in fruits, the history of pitless fruits, and the advantages and disadvantages of the presence of stone in fruit. It is already known that plants have evolved a variety of seed protection and dispersal strategies. Stone-fruit-bearing species have evolved a one-of-a-kind adaptation in which the seed is encased in an extremely hard wood-like shell known as the stone. The stone is produced by the lignification of the fruit endocarp layer, which differentiates drupes from other plants. After programmed cell death and the deposition of cellulose and lignin in the secondary cell wall, stone cells arise from parenchyma cells.

Different genes have different roles in fruits [56]; therefore, it is very important to fully understand the mechanism of stone or hardened endocarp formation in fruits. Unfortunately, this has not received much scientific attention in the past. Studies on stone formation are very poorly elicited and there is little scientific information available about the important processes of the endocarp, including anatomical observations. For a more complete picture of gene expression, biochemical analyses are, therefore, required to be performed. Furthermore, assessing the gene expression of multiple TFs (transcription factors) participating in endocarp cell production should be used to establish the time required for endocarp formation. According to some studies, fruit stones contain a high amount of lignin, hence genes should be targeted by genetic engineering to minimize the level of lignification. Finding out how they obtain so much more lignin could also lead to new strategies to improve the quality of tree wood or produce energy-dense biofuel crops.

## Figures and Tables

**Table 1:** Molecular regulation of endocarp and lignin formation in fruits.

S.No	Plant Species	Parts	Genes	TFs	References
1	Apricots	**Endocarp**	*PAL, C4H, CCoAOMT, COMT, C3’H, F5H, 4CLCCR, and CAD*	NST1	[33]
2	Peach		*PpLAC20, PpLAC30*	NAC and MYB	[4,7]
3	*E. japonica*	**Lignin**	-----	EjMYB1 and EjMYB2	[40,49]
4	*E. grandis*		-----	EgMYB2	[48]
5	Peach		-----	SHATTERPROOF, SEEDSTCK, and NAC SECONDARY WALL THICKENING PROMOTING FACTOR 1	[45,46]
6	Poplar		-----	PtoMYB92/PtrMYB3/PtrMYB20	
7	*P. taeda*		-----	PtMYB1 and PtMYB8	[47]

## Data Availability

Not applicable.
